# Efficacy of pulsed electromagnetic field therapy on acute radiodermatitis in breast cancer patients: a randomized controlled study

**DOI:** 10.1007/s00520-026-10322-9

**Published:** 2026-02-05

**Authors:** Mahmoud Hamada Mohamed, Mohamed N. Selim, Marwa Elsayed Mohamed, Mohamed Magdy Elmeligie, Ahmed Mahmoud Kadry, Reda Kotb Abdelrazik, Sara Mohamed Samir, Roshdy Mohamed Kamel, Kamaleldin Ahmed kamal, Mahmoud Elshazly

**Affiliations:** 1 Physical Therapy for Integumentary Department, Faculty of Physical Therapy, Benha University, Benha, 13511 Egypt; 2 Basic Science Department, Faculty of Physical Therapy, Beni-Suef University, Beni Suef, Egypt; 3 Physical Therapy for Women and Child Health Department, Faculty of Physical Therapy, Beni Suef University, Beni Suef, Egypt; 4 Basic Science Department, Faculty of Physical Therapy, Alhayah University, Cairo, Egypt; 5 Physical Therapy for Integumentary Department, Faculty of Physical Therapy, Kafr Elsheikh University, Kafr Elsheikh, Egypt; 6 Physical Therapy for Musculoskeletal Disorders and Its Surgery Department, Faculty of Physical Therapy, Benha University, Benha, Egypt; 7 Physical Therapy for Musculoskeletal Disorders and Its Surgery Department, Faculty of Physical Therapy, Cairo University, Cairo, Egypt; 8 Basic Sciences Department, Faculty of Physical Therapy, Benha University, Benha, Egypt; 9 Physical Therapy for Surgery Department, Faculty of Physical Therapy, South Valley University, South Valley, Egypt

**Keywords:** Pulsed electromagnetic field, Hypo-fractionated whole breast irradiation, Sonography, Radiation Therapy Oncology Group

## Abstract

**Purpose:**

The objective of this research was to synthesize the evidence about the usage of pulsed electromagnetic field (PEMF) in women with breast cancer undergoing hypo-fractionated whole-breast irradiation (HFWBI) for the treatment of acute radiodermatitis.

**Methods:**

The study included 50 patients (35–55 years) with unilateral breast cancer post-lumpectomy who received HFWBI. They were randomized into Group A (PEMF plus standard skin care) and Group B (standard skin care only). Skin reactions were assessed during PEMF therapy using sonographic skin thickness measurements and RTOG criterion.

**Results:**

After radiation, skin thickness increased in both groups, peaking at 4 weeks (PEMF 2.82 mm; control 2.74 mm, *p* = 0.030). By 6 weeks, thickness declined in the PEMF group but remained high in controls (*p* < 0.001), and by 8 weeks PEMF returned near baseline (2.01 mm) while controls stayed thickened (*p* < 0.001). Skin reactions worsened in both groups by week 4, with more severe cases in controls (36% vs. 12%, *p* = 0.150). By week 6, PEMF patients mostly had mild reactions (72% Grade 0–1) compared to predominantly severe reactions in controls (84% Grade 2–3, *p* = 0.001). At 8 weeks, recovery was greater with PEMF, as 52% returned to Grade 0 versus only 8% in controls (*p* = 0.001).

**Conclusion:**

Results such as these indicate that PEMF therapy accelerates recovery from radiotherapy and diminishes the intensity of acute radiodermatitis.

**Trial registration:**

Trial registration number: NCT06003764. Date of registration: 12 August 2023. Prospectively registered for prospectively registered trials.

## Introduction

Acute radiodermatitis (ARD) is an unpleasant and irritating skin reaction that develops either immediately or weeks once initiating radiotherapy, with a prevalence of 98% in women with breast cancer [1]. Radiation therapy (RT) specifically targets neoplastic cells and causes DNA with double strands to break down, which results in cell destruction and apoptosis, while also adversely affecting adjacent healthy tissue [2]. The skin’s quick turnover of tissue renders it more susceptible to the deleterious impacts of radiation; ARD is a prevalent adverse effect, varying from minor erythema to wet desquamation; more serious cases may result in ulceration and necrosis [3].

ARD begins with erythema followed by dry desquamation (grade 1). Tender or brilliant erythema is the characteristic of a grade 2 A skin response. A grade 2B patient has irregular moist desquamation in skin creases, as well as minor edema. In severe situations, wet desquamation beyond the skin fold may be accompanied by pitting oedema (grade 3) [4, 5].

The impact of radiodermatitis is wide and covers diminished life quality resulting from suffering and pain, reduced self-esteem, suspension of radiotherapy and infection, and hence limiting neoplasm management [6].

In general, wounds and inflammatory tissues in the human body are associated with oxygen deficiency and inadequate circulation of blood. Low frequency pulsed magnetic treatment was commonly utilized to increase metabolism and generate detoxification. Magnetic therapy generates mild electrical currents within the body’s tissues, which increases cell surface capacity. This technique improves blood flow, nutrient delivery effectiveness, and the elimination of waste products from the damaged tissues [7].

Magnetic fields are additionally utilized as natural pain relievers to encourage healing and regeneration and relieve stiffness, edema, and acidity from wounds. One study indicated that applying a magnetic field to the area around a joint accelerated bone healing by increasing collagen concentration and stimulating Ca2 + migration to the location of a lesion [8].

At the level of molecules, pulsed magnetic treatment appears to modify multiple cytokines, including interleukin from macrophages and lymphocytes. PEMF’s anti-inflammatory effect was additionally documented through regulating the release of cytokines that induce inflammation (TNF-a, IL-6, and IL-8) and improved anti-inflammatory cytokine synthesis (IL-10) [9].

## Materials and methods

### Trial design

This is a randomized controlled study involving 50 females who had been diagnosed with breast cancer and were experiencing acute radiodermatitis. The participants were involved in the study for a duration of 2 months. Before any experimental procedure began, each participant provided written informed consent. After examinations, individuals were randomly allocated to the study group, which received PEMF alongside traditional skin care, and the control group, which received traditional skin care. Evaluations were carried out pretreatment and every two weeks of treatment for 8 consecutive weeks.

### Ethical approval

The human-use study adhered to all relevant national regulations and institutional standards, complied with the requirements of the Declaration of Helsinki, and received approval from the ethical council of the Physical Therapy Faculty at the University of South Valley (P.T-SUR-08/2023–518). The research trial has been registered on ClinicalTrials.gov (gov ID: NCT06003764) prior to recruitment. Every participant gave written informed consent and were advised of their freedom to withdraw at any moment without penalties.

### Randomization and blinding

Participants were directed to Ahram Canadian University’s faculty of physical therapy out-patient clinic, where the main investigator presented to everyone who participated an in-depth explanation about the treatment methods. Each participant provided written informed permission, and it was made clear that they could withdraw from joining the research at any moment without consequences. All data had codes to ensure the confidentiality and anonymity of any collected information.

An unbiased and blinded research assistant randomly allocated individuals with ARD to one of two groups. The assistant opened sealed envelopes filled with random cards, which are produced by computers. The participants were concealed from the study group they were assigned. Also, they were blinded to the type of treatment applications they received (PEMF or control group). Furthermore, the radiologist who performed the skin thickness measurement was blinded to the patients’ group allocation.

### Participant

The study work was completed between November 2023 and June 2025. Participants were chosen by referrals from oncologists in national cancer institute in Cairo, Egypt. Patients were checked for eligibility at Ahram Canadian University’s outpatient clinic. The patient’s age ranged from 35 to 55. The patients were separated randomly into two groups, and they classified 2 groups of similar numbers using a one-to-one method: The experimental group (25 patients) had pulsed electromagnetic therapy along with traditional skin care, whereas the control group (25 patients) had only traditional skin care. Every individual in both groups got the same medical care. Oncology staff supervised medical procedures, to ascertain the illness and establish criteria for inclusion and exclusion.

The included criteria are those who were diagnosed with primary unilateral breast cancer and had hypo-fractionated whole breast radiation (HFWBI) following lumpectomy, and their age was between 35 and 55 years. They were scheduled to undergo radiation treatment, including doses of 40–42.5 Gy delivered in 15–16 fractions weekly within a duration of roughly 3 weeks [10].

Exclusive criteria were previous irradiation to the exact same female breast, bilateral breast cancer, metastatic condition, mastectomy, infections, and simultaneous chemotherapy, as well as those with pacemakers and patients suffering from open wounds or already existing skin disorders in the treatment region [11].

### Treatment intervention

#### First group (PEMF group)

We used the PHYSIOMED MAG-Expert® (Made in Germany); it is a pulsed electromagnetic field therapy (PEMFT) device, its system designed for medical and rehabilitation applications. It operates on a 230 V AC power supply, with a maximum consumption of 410 VA, and delivers field strengths up to 100 Gauss across a frequency range of 1–100 Hz. The system allows for both continuous and pulsed modes tailored to specific clinical conditions [12].

Our study treatment parameters adjusted to low-frequency 15 Hz, 50% intensity, pulsed (5 s/min cycle) and 20 min/session, ensuring localized anti-inflammatory and regenerative effects without direct skin contact, which is advantageous in sensitive or inflamed dermal conditions.

The PEMF was applied from the first day of radiotherapy, immediately after the first radiation session. The patient comfortably rests in the plinth for twenty minutes while keeping their breast within the magnetic field of the coil structure, twice per week every 3 days for eight continuous weeks.

Also all patients receive traditional skin care. From the first day of RT, patients obtained hospital conventional skin care, including a 3-day application of hydroactive colloid gel. An absorbent, foam, self-adhesive silicone bandage was utilized to treat a painful cutaneous reaction or wet desquamation. Additionally, the patients were also instructed to adhere to standard skin care protocols, such as washing gently using light soap or without it and tapping dry with an absorbent towel rather than rubbing [13].

#### The second group (controlled group)

In this group all patients receive traditional skin care. From the first day of RT, patients obtained hospital conventional skin care, including a three-day application of hydroactive colloid gel. An absorbent, foam, self-adhesive silicone bandage was utilized to treat a painful cutaneous reaction or wet desquamation. Additionally, the patients were also instructed to adhere to standard skin care protocols, such as washing gently using light soap or without it and tapping dry with an absorbent towel rather than rubbing [13].

### Patient evaluation

#### Outcome measures

## Primary outcome measure: ultrasonography

Skin thickness can be effectively assessed with ultrasound, with strong intra- and inter-observer reliability, multiple research investigations indicate that ultrasonography can detect the level of ARD by assessing an elevation in the thickness of skin within the treated site [14]. Some researchers have utilized the normal elasticity percentage for skin tissue following radiation to determine the buildup of fluid in the layers of skin [15].

The machine (L15–4 linear-array transducer; Supersonics Aixplorer; Supersonic Imagine; France was applied to assess skin thickness. Ultrasonography measures were performed by the same radiologist for both research groups throughout the testing process. To improve effectiveness, an ultrasonic gel with a 1 mm thickness was utilized to serve as a connecting medium. To improve accuracy, the average of three measurements taken from the most clinically affected skin region of the breast irradiated area was recorded. Special care was taken to avoid any pressure on the skin surface, and the probe is gently applied to the surface [16].

## Secondary outcome measure: Radiation Therapy Oncology Group (RTOG) SCALE

The RTOG (Radiation Therapy Oncology Group) index is applied for describing skin reaction. We sought a description of the degree of skin irritation so that we could allocate it to the appropriate location on the rating system as follows:

RTOG 0, no change; RTOG 1 shows minor erythema, sweating, epilation, and dry desquamation. RTOG 2, patchy wet desquamation, painful or bright erythema, and significant oedema; RTOG 3, pitting oedema and moist desquamation in areas beyond skin creases; RTOG 4: bleeding, necrosis, and ulcers; RTOG 5: Mortality [17, 18].

### Calculation of sample size

To determine the minimum number of experiments required, an initial power calculation was conducted by G*Power software (version 3.1.9.7). At an acceptable level of significance of 0.05, 44 participants (22 per group) would be enough to detect a significant effect size (*f* = 0.4) with 80% power. To strengthen statistical power and ensure feasibility, the sample size was increased to 50 participants, with 25 in each group.

### Statistical analysis

IBM SPSS Statistics, edition 26, was used to investigate the data. Descriptive statistics were provided as mean ± SD or median (interquartile range, IQR) where applicable. The Shapiro–Wilk test and a visual evaluation of histograms were used to ensure data distribution was typical. Non-parametric tests were utilized since the data were not grouped in a regular pattern. To compare continuous data (skin thickness at every single point) between groups, the Mann–Whitney *U* test was used. To analyze variations within groups over time, the Friedman test was used, followed by Wilcoxon signed-rank tests to compare pairs. A categorical variable (RTOG grades) was summarized using percentages and frequencies. Variations among groups were analyzed with the Chi-square test or Fisher’s exact test, when suitable. A *p*-value < 0.05 indicated statistical significance.

## Results

**Fig. 1:**
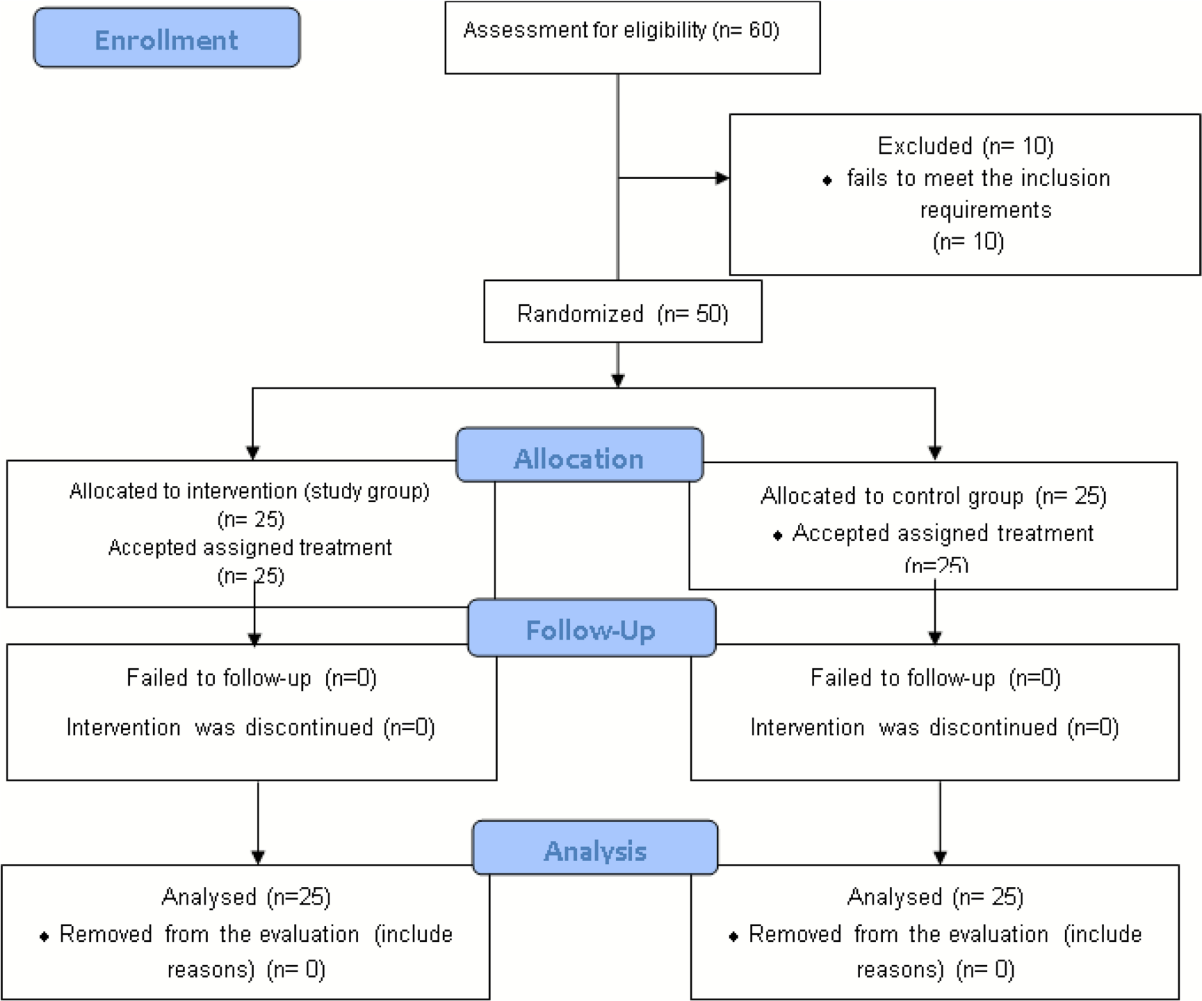
Eligibility chart

Our initial evaluation used an intent-to-treat strategy, including all randomized subjects. A whole group of 60 ladies were eligible to compete. The last analysis of statistics involved 50 patients, with 25 matched into the PEMF group and 25 to the standard skin care group (Fig. 1).

At baseline, there existed no statistically significant difference in BMI or age between the two groups (Table 1).

**Table 1:** General features of subjects in the tested groups

Variables	Groups (Mean ± SD)		*p*-value
	Group A	Group B	
Age (year)	42.79 ± 6.11	41.45 ± 4.69	0.532
Weight (kg)	72.28 ± 9.23	73.26 ± 5.32	0.623
Height (cm)	161.13 ± 4.88	159.07 ± 3.69	0.961
BMI (kg/m^2^)	30.24 ± 1.83	29.55 ± 1.27	0.298

*SD* standard deviation; *p*-value, level of significance

### Primary outcome measure results

**Fig. 2:**
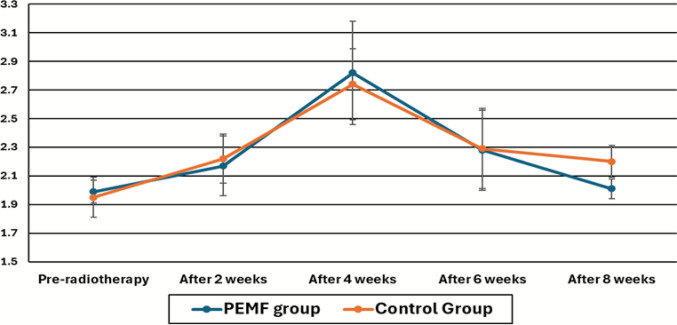
Timeline of skin thickness changes during and after radiotherapy in PEMF and control groups

In PEMF group, thickness of the skin considerably increased from the baseline to 2 weeks, indicating the anticipated early radiation-induced oedema. Subsequently, notable reductions were recorded between 2–4 weeks, 4–6 weeks, and 6–8 weeks, demonstrating an overall enhancement from 2 to 8 weeks, signifying a gradual recovery trajectory towards baseline levels. Conversely, the control group exhibited a significant progressive increase in skin thickness, observed between weeks 2 and 4 and again between weeks 4 and 6., confirming the progressive build-up of acute edema. However, no significant improvement was observed between 6 and 8 weeks or from 2 to 8 weeks, suggesting a more prolonged and persistent swelling in the absence of PEMF therapy (Table 2 and Fig. 2).

**Table 2:** Skin thickness (mm) at different time points in PEMF (Group A) and control (Group B)

Time point		Group A (PEMF, ***n*** = 25)	Group B (Control, ***n*** = 25)	*p*-value
Pre-radiotherapy	Mean ± SD	1.99 ± 0.14	1.95 ± 0.08	> 0.999
	**Median (IQR)**	2.00 (0.15)	2.00 (0.10)	
After 2 weeks	Mean ± SD	2.17 ± 0.17	2.22 ± 0.21	0.735
	**Median (IQR)**	2.20 (0.30)	2.20 (0.35)	
After 4 weeks	Mean ± SD	2.82 ± 0.25	2.74 ± 0.36	0.030*
	**Median (IQR)**	2.80 (0.40)	2.80 (0.50)	
After 6 weeks	Mean ± SD	2.28 ± 0.28	2.29 ± 0.28	< 0.001*
	**Median (IQR)**	2.20 (0.45)	2.20 (0.30)	
After 8 weeks	Mean ± SD	2.01 ± 0.11	2.20 ± 0.07	< 0.001*
	**Median (IQR)**	2.00 (0.11)	2.20 (0.15)	

Between-group comparisons of skin thickness at each time point were performed using the Mann–Whitney U test

### Secondary outcome measure results

**Fig. 3:**
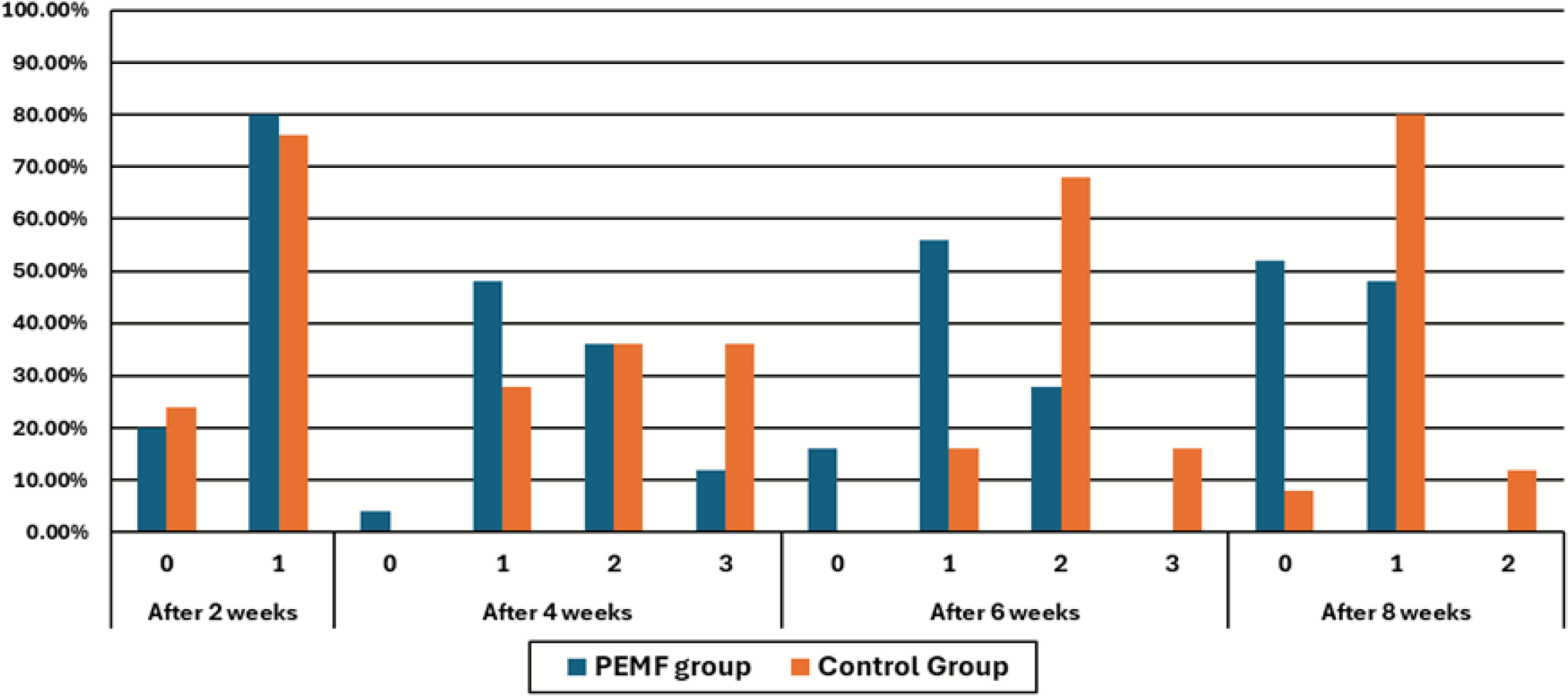
Distribution of RTOG grades across follow-up in PEMF and control groups

At two weeks post-radiotherapy, most of the patients in the study had mild responses (RTOG Grade 1), with no meaningful difference between the PEMF and control groups (*p* > 0.999). By 4 weeks, skin reactions had progressed in both groups, with most patients developing Grade 1–2 dermatitis. Notably, a greater number of patients in the controlled group reached 3rd grade severity (36.0% vs. 12.0% in PEMF), but this difference could not achieve statistical significance. (*p* = 0.150). This suggests that the peak of acute radiodermatitis occurred around week 4 in both groups. At 6 weeks, a clear divergence between groups became evident. In the PEMF group, most patients had milder reactions (72% remained at Grade 0–1), while the majority of the control group showed more severe reactions (68% Grade 2 and 16% Grade 3). PEMF therapy effectively protects against severe radiodermatitis (*p* = 0.001). By 8 weeks, recovery was observed in both groups. However, significantly more patients in the PEMF group had returned to Grade 0 (52% vs. 8%), although the population control reported higher frequencies of sustained Grade 1–2 toxicity. The difference remained significant (*p* = 0.001) (Tables 3 and 4 and Fig. 3).

**Table 3:** Distribution of RTOG grading at different follow-up intervals in PEMF (Group A) versus control (Group B)

Time point	RTOG grade	Group A (*n* = 25)	Group B (*n* = 25)	*p*-value
After 2 weeks	0	5 (20.0%)	6 (24.0%)	> 0.999
	1	20 (80.0%)	19 (76.0%)	
After 4 weeks	0	1 (4.0%)	0 (0.0%)	0.150
	1	12 (48.0%)	7 (28.0%)	
	2	9 (36.0%)	9 (36.0%)	
	3	3 (12.0%)	9 (36.0%)	
After 6 weeks	0	4 (16.0%)	0 (0.0%)	0.001*
	1	14 (56.0%)	4 (16.0%)	
	2	7 (28.0%)	17 (68.0%)	
	3	0 (0.0%)	4 (16.0%)	
After 8 weeks	0	13 (52.0%)	2 (8.0%)	0.001*
	1	12 (48.0%)	20 (80.0%)	
	2	0 (0.0%)	3 (12.0%)	

Values are presented as *n* (%). Comparisons between groups at each time point were performed using the Chi-square test or Fisher’s exact test, as appropriate. A *p*-value < 0.05 was considered statistically significant

**Table 4:** Within-group changes in RTOG scores across follow-up periods (Friedman and Wilcoxon signed-rank tests)

Group	Friedman Test (χ^2^, df = 3)	*p*-value	Pairwise comparison	Z-value	*p*-value
Group A (PEMF)	32.18	< 0.001	2w vs 4w	−3.578	< 0.001*
			4w vs 6w	−2.524	0.012*
			6w vs 8w	−3.578	< 0.001*
			2w vs 8w	−2.138	0.033*
Group B (Control)	47.96	< 0.001	2w vs 4w	−4.005	< 0.001*
			4w vs 6w	−0.426	0.670
			6w vs 8w	−4.347	< 0.001*
			2w vs 8w	−2.111	0.035*

Within-group changes over time were assessed using the Friedman test, followed by Wilcoxon signed-rank tests for pairwise comparisons. *p ≤ 0.05 considered significant (Bonferroni-adjusted)

## Discussion

From previous results, the within-group analysis demonstrated distinct temporal patterns of skin thickness in both treatment arms. In the PEMF group, thickness increased significantly from 2 to 4 weeks, corresponding to the expected peak of radiation-induced edema. This was followed by significant reductions between 4 and 6 weeks and again between 6 and 8 weeks, with an overall improvement from 2 to 8 weeks, indicating a clear recovery trajectory toward baseline.

Conversely, the control group exhibited a significant progressive increase in skin thickness, observed between weeks 2 and 4 and again between weeks 4 and 6., confirming the progressive build-up of acute edema. However, no significant improvement was observed between 6 and 8 weeks or from 2 to 8 weeks, suggesting a more prolonged and persistent swelling in the absence of PEMF therapy.

Taken together, these findings support the role of PEMF in facilitating earlier resolution of radiation-induced skin edema, with a recovery phase evident by week 6, compared with a delayed or incomplete resolution in controls and the timeline of mean skin thickness (mm) over time in PEMF and control groups, showing peak thickening at 4 weeks with faster recovery in the PEMF group.

The magnetic fields have the ability to travel through cellular tissue and trigger deep tissues without invasive or contact tools. PEMF, as opposed to static magnetic fields or constant fixed-intensity stimulation, transmits signals through tissue efficiently while reducing threshold-related disturbances. Previous research has shown that PEMF stimulation can accelerate skin wound healing, tissue repair, vascular rebuilding, and anti-inflammatory abilities [19, 20].

Tumor necrosis factor‐α (TNF‐α), a cytokine produced primarily by activated macrophages, is a central mediator of systemic inflammatory responses. Exposure to PEMF has been demonstrated to substantially reduce TNF‐α concentrations. Because PEMF signals can rapidly permeate all layers of the targeted tissue, they are capable of modulating physiological anti-inflammatory pathways, thereby facilitating subsequent processes of tissue healing [21].

Significantly, PEMF exhibited a level of selectivity for malignant cells while preserving normal tissues in certain experimental conditions. Administration of optimized PEMF conditions markedly diminished the viability of MDA-MB-231 breast cancer tissue while preserving the integrity of normal epithelial cells. This selectivity emphasizes PEMF’s ability to act as a complementary therapeutic technique alongside traditional therapies [22, 23].

The findings of this research align with a study involving hairless mice with chemically induced atopic dermatitis, which showed that PEMF at 15 Hz and 75 Hz diminished skin inflammation and lesion size relative to controls, indicating that PEMF initiates electrochemical responses that influence the body’s immune system [24, 25].

PEMT has been therapeutically employed to facilitate wound closure, enhance the healing of chronic ulcers, and stimulate growth and circulation. PEMT stimulates capillary vessel development and endothelial cell growth, leading to increased release of IGF and TGF-β, this technique speeds up the healing process and is essential at all stages. Furthermore, it has been shown that it excites mitochondria ribosomes and hence enhances enzyme function [26].

Additionally, it increases the amount of oxygen in the body and helps remove harmful substances from tissues. PEMF therapy works by using low-voltage electric impulses to activate muscles and nerves, and it has special healing capabilities, which may eliminate infections, minimize pain, boost immunity, improve the flow of lymph, and help control blood flow and body processes by encouraging the growth of new blood vessels [27].

This outcome is congruent with the work of Goudarzi et al., who discovered the PEMF treatment increased the biomechanical ability of healed diabetic wounds on day 27 post-wounding and claimed that this benefit could be attributed to higher collagen fiber number and better collagen fibril arrangement or maturation [28].

## Strength and limitations

The randomized, controlled design of this trial and the radiologist’s blinding of the objective primary outcome measure (skin thickness) are two of its strength points. However, there are other limitations. The single-center design may limit the results’ applicability to a variety of patient groups, even if the study’s sample size of 50 patients (25 in each group) was statistically adequate based on our sample size calculation. Furthermore, the eight-week trial length was adequate to evaluate the impact of PEMF on acute radiodermatitis and early recovery. Therefore, to assess the effect of PEMF therapy on long-term radiation-induced toxicities, such as fibrosis, we recommend additional multicenter studies with a larger sample size and longer PEMF therapy duration. We also suggest more in vivo research that may provide light on the underlying physiological mechanisms of PEMF in the management of radiodermatitis. Additionally, future research using various PEMF parameters would aid in determining the ideal dosage for the treatment of acute radiodermatitis.

## Conclusion

According to the findings of this single-blind, random, control study, pulsed electromagnetic field therapy had a substantial effect on acute radiodermatitis in breast cancer patients. Researchers feel that this evidence could have a significant impact on the development of novel radiodermatitis treatments.

## Data Availability

Data is available upon reasonable request from corresponding author.

## References

[1] Bontempo PSM, Ciol MA, Menêses AG, Simino GPR, Ferreira EB, Reis P (2021) Acute radiodermatitis in cancer patients: incidence and severity estimates. Rev Esc Enferm USP 55:e03676 33886907 10.1590/S1980-220X2019021703676

[2] Leventhal J, Young MR (2017) Radiation dermatitis: recognition, prevention, and management. Oncology (Williston Park) 31(12):885–7, 94-9 29297172

[3] Ramseier JY, Ferreira MN, Leventhal JS (2020) Dermatologic toxicities associated with radiation therapy in women with breast cancer. Int J Womens Dermatol 6(5):349–356 33898697 10.1016/j.ijwd.2020.07.015 PMC8060663

[4] Fishbourne E, Ludi AB, Wilsden G, Hamblin P, Statham B, Bin-Tarif A et al (2017) Efficacy of a high potency O(1) Manisa foot-and-mouth disease vaccine in cattle against heterologous challenge with a field virus from the O/ME-SA/Ind-2001 lineage collected in North Africa. Vaccine 35(20):2761–2765 28396208 10.1016/j.vaccine.2017.02.047

[5] Borm KJ, Loos M, Oechsner M, Mayinger MC, Paepke D, Kiechle MB et al (2018) Acute radiodermatitis in modern adjuvant 3D conformal radiotherapy for breast cancer - the impact of dose distribution and patient related factors. Radiat Oncol 13(1):218 30404664 10.1186/s13014-018-1160-5 PMC6223003

[6] Fuzissaki MA, Paiva CE, Oliveira MA, Lajolo Canto PP, Paiva Maia YC (2019) The impact of radiodermatitis on breast cancer patients’ quality of life during radiotherapy: a prospective cohort study. J Pain Symptom Manage 58(1):92-99.e1 30974233 10.1016/j.jpainsymman.2019.03.017

[7] Öcal I, Kalkan T, Günay İ (2008) Effects of alternating magnetic field on the metabolism of the healthy and diabetic organisms. Braz Arch Biol Technol 51:523–530

[8] Ross CL, Siriwardane M, Almeida-Porada G, Porada CD, Brink P, Christ GJ et al (2015) The effect of low-frequency electromagnetic field on human bone marrow stem/progenitor cell differentiation. Stem Cell Res 15(1):96–108 26042793 10.1016/j.scr.2015.04.009 PMC4516580

[9] Vergallo C, Dini L, Szamosvölgyi Z, Tenuzzo BA, Carata E, Panzarini E et al (2013) In vitro analysis of the anti-inflammatory effect of inhomogeneous static magnetic field-exposure on human macrophages and lymphocytes. PLoS ONE 8(8):e72374 23991101 10.1371/journal.pone.0072374 PMC3753352

[10] Gupta A, Ohri N, Haffty BG (2018) Hypofractionated radiation treatment in the management of breast cancer. Expert Rev Anticancer Ther 18(8):793–803 29902386 10.1080/14737140.2018.1489245 PMC6312641

[11] Robijns J, Censabella S, Claes S, Pannekoeke L, Bussé L, Colson D et al (2018) Prevention of acute radiodermatitis by photobiomodulation: a randomized, placebo-controlled trial in breast cancer patients (TRANSDERMIS trial). Lasers Surg Med. 10.1002/lsm.22804 10.1002/lsm.22804 29427390

[12] AG. PE (2025) MAG-Expert® with cylinder Ø 30 cm [Available from. https://physiomed.de/en/produkt/mag-expert-mit-zylinder-o-30-cm. Accessed 1 Sept 2025

[13] Censabella S, Claes S, Orlandini M, Braekers R, Thijs H, Bulens P (2014) Retrospective study of radiotherapy-induced skin reactions in breast cancer patients: reduced incidence of moist desquamation with a hydroactive colloid gel versus dexpanthenol. Eur J Oncol Nurs 18(5):499–504 24877859 10.1016/j.ejon.2014.04.009

[14] Polańska A, Silny W, Jenerowicz D, Knioła K, Molińska-Glura M, Dańczak-Pazdrowska A (2015) Monitoring of therapy in atopic dermatitis–observations with the use of high-frequency ultrasonography. Skin Res Technol 21(1):35–40 24894324 10.1111/srt.12153

[15] Schack LH, Alsner J, Overgaard J, Andreassen CN, Offersen BV (2016) Radiation-induced morbidity evaluated by high-frequency ultrasound. Acta Oncol 55(12):1498–1500 27581725 10.1080/0284186X.2016.1222079

[16] Adriaenssens N, Belsack D, Buyl R, Ruggiero L, Breucq C, De Mey J et al (2012) Ultrasound elastography as an objective diagnostic measurement tool for lymphoedema of the treated breast in breast cancer patients following breast conserving surgery and radiotherapy. Radiol Oncol 46(4):284–295 23412910 10.2478/v10019-012-0033-z PMC3572897

[17] Kole AJ, Kole L, Moran MS (2017) Acute radiation dermatitis in breast cancer patients: challenges and solutions. Breast Cancer (Dove Med Press) 9:313–323 28503074 10.2147/BCTT.S109763 PMC5426474

[18] Cox JD, Stetz J, Pajak TF (1995) Toxicity criteria of the Radiation Therapy Oncology Group (RTOG) and the European Organization for Research and Treatment of Cancer (EORTC). Int J Radiat Oncol Biol Phys 31(5):1341–1346 7713792 10.1016/0360-3016(95)00060-C

[19] Athanasiou A, Karkambounas S, Batistatou A, Lykoudis E, Katsaraki A, Kartsiouni T et al (2007) The effect of pulsed electromagnetic fields on secondary skin wound healing: an experimental study. Bioelectromagnetics 28(5):362–368 17486634 10.1002/bem.20303

[20] Costantini E, Sinjari B, D’Angelo C, Murmura G, Reale M, Caputi S (2019) Human gingival fibroblasts exposed to extremely low-frequency electromagnetic fields: in vitro model of wound-healing improvement. Int J Mol Sci. 10.3390/ijms20092108 31035654 10.3390/ijms20092108 PMC6540598

[21] Vinhas A, Rodrigues MT, Gonçalves AI, Reis RL, Gomes ME (2020) Pulsed electromagnetic field modulates tendon cells response in IL-1β-conditioned environment. J Orthop Res 38(1):160–172 31769535 10.1002/jor.24538

[22] Tai YK, Chan KKW, Fong CHH, Ramanan S, Yap JLY, Yin JN et al (2021) Modulated TRPC1 expression predicts sensitivity of breast cancer to doxorubicin and magnetic field therapy: segue towards a precision medicine approach. Front Oncol 11:783803 35141145 10.3389/fonc.2021.783803 PMC8818958

[23] Alcantara DZ, Soliman IJS, Pobre RF, Naguib RNG (2017) Effects of pulsed electromagnetic fields on breast cancer cell line MCF 7 using absorption spectroscopy. Anticancer Res 37(7):3453–3459 28668834 10.21873/anticanres.11713

[24] Kim JY, Lee JY, Lee JW, Lee SK, Park CS, Yang SJ et al (2022) Evaluation of atopic dermatitis improvement caused by low-level, low-frequency pulsed electromagnetic fields. Bioelectromagnetics 43(4):268–277 35476222 10.1002/bem.22405

[25] Kim J-Y, Hong J-E, Woo S-H, Rhee K-J, Kim YS, Lee Y-H (2024) Effect of pulsed electromagnetic field stimulation on splenomegaly and immunoglobulin E levels in 2, 4-dinitrochlorobenzene-induced atopic dermatitis mouse model. Appl Sci. 10.3390/app1414634639027034

[26] Keskin Y, Taştekin N, Kanter M, Top H, Özdemir F, Erboğa M et al (2019) The effect of magnetic field therapy and electric stimulation on experimental burn healing. Turk J Phys Med Rehabil 65(4):352–360 31893272 10.5606/tftrd.2019.2899 PMC6935725

[27] Tabakan I, Yuvacı AU, Taştekin B, Öcal I, Pelit A (2022) The healing effect of pulsed magnetic field on burn wounds. Burns 48(3):649–653 34670708 10.1016/j.burns.2021.06.001

[28] Goudarzi I, Hajizadeh S, Salmani ME, Abrari K (2010) Pulsed electromagnetic fields accelerate wound healing in the skin of diabetic rats. Bioelectromagnetics 31(4):318–323 20082338 10.1002/bem.20567

